# Effect of cold stress on photosynthetic physiological characteristics and molecular mechanism analysis in cold-resistant cotton (ZM36) seedlings

**DOI:** 10.3389/fpls.2024.1396666

**Published:** 2024-05-13

**Authors:** Youzhong Li, Jincheng Zhu, Jianwei Xu, Xianliang Zhang, Zongming Xie, Zhibo Li

**Affiliations:** ^1^ College of Agriculture, Shihezi University, Shihezi, Xinjiang, China; ^2^ Cotton Research Institute, Xinjiang Academy of Agricultural and Reclamation Science/Xinjiang Production and Construction Group Key Laboratory of Crop Germplasm Enhancement and Gene Resources Utilization, Shihezi, Xinjiang, China; ^3^ Xinjiang Production and Construction Group Key Laboratory of Crop Germplasm Enhancement and Gene Resources Utilization, Biotechnology Research Institute, Xinjiang Academy of Agricultural and Reclamation Sciences, Shihezi, Xinjiang, China; ^4^ Western Research Institute, Chinese Academy of Agricultural Sciences (CAAS), Changji, China

**Keywords:** cotton, low-temperature stress, photosynthetic parameters, transcriptomic analysis, DEGs, VIGS

## Abstract

Low temperature and cold damage seriously hinder the growth, development, and morphogenesis of cotton seedlings. However, the response mechanism of cotton seedlings under cold stress still lacks research. In this study, transcriptome sequencing, gas exchange parameters, and rapid chlorophyll fluorescence parameters were analyzed in leaves of cold-tolerant upland cotton variety “ZM36” under different temperature stress [25°C (T25, CK), 15°C (T15), 10°C (T10), and 4°C (T4)]. The results showed that the net photosynthetic rate (Pn), stomatal conductance (Gs), transpiration rate (Tr), PSII potential maximum photochemical efficiency (Fv/Fm), and performance index (PIabs) of cotton leaves significantly decreased, and the intercellular CO_2_ concentration (Ci) and Fo/Fm significantly increased under cold stress. The transcriptome sequencing analysis showed that a total of 13,183 DEGs were involved in the response of cotton seedlings at each temperature point (T25, T15, T10, and T4), mainly involving five metabolic pathways—the phosphatidylinositol signaling system, photosynthesis, photosynthesis antenna protein, carbon fixation in photosynthetic organisms, and carotenoid synthesis. The 1,119 transcription factors were discovered among all the DEGs. These transcription factors involve 59 families, of which 15.8% of genes in the NAC family are upregulated. Through network regulatory analysis, the five candidate genes *GhUVR8* (*GH_A05G3668*), *GhPLATZ* (*GH_A09G2161*), *GhFAD4-1* (*GH_A01G0758*), *GhNFYA1* (*GH_A02G1336*), and *GhFAD4-2* (*GH_D01G0766*) were identified in response to cold stress. Furthermore, suppressing the expression level of *GhPLATZ* by virus-induced gene silencing led to the reduction of low temperature resistance, implying *GhPLATZ* as a positive regulator of low temperature tolerance. The findings of the study revealed a piece of the complex response mechanism of the cold-tolerant variety “ZM36” to different cold stresses and excavated key candidate genes for low temperature response, which provided support for accelerating the selection and breeding of cotton varieties with low temperature tolerance.

## 1 Introduction

Cotton (*Gossypium hirsutum* L.) is sensitive to temperature during its growth and development (Shan et al., 2007). Xinjiang is the main cotton-producing region in China. In the past 40 years, the frequency of cotton cultivation in Xinjiang has been 30% under cold stress. The frequency of “late spring cold” weather especially is very high, which leads to hindered growth, decreased uniformity, and delayed growth and development of cotton seedlings, greatly affecting the yield and quality of cotton (Rihan et al., 2017; Li et al., 2020a). Therefore, in-depth analysis of the response mechanism of cotton seedlings to low temperature cold damage is of great significance to improve their cold tolerance.

The adaptation mechanism of plants to cold stress involves complex and intricate physiological and molecular regulatory networks (Raju et al., 2018; Kidokoro et al., 2022). On the physiological level, it manifests as decreased enzyme activity, damage to the membrane system, decreased photosynthetic efficiency, and cell dehydration. At the molecular level, the most extensively studied pathway is the DREB/CBF low temperature stress regulation pathway (Chinnusamy et al., 2007), which can bind to the A/GCCGAC dehydration response element (DRE) in the low temperature response gene (*COR*) promoter, thereby activating the expression of the *COR* gene. The *COR* gene encodes a developmental stress protein (LEA), which can enhance plant tolerance to low temperature, dehydration, or abscisic acid stress (Yamaguchi-Shinozaki and Shinozaki, 1994; Stockinger et al., 1997). Simultaneously, some transcription factor family members also play key roles in cold stress, such as AP2/ERF, NAC, bHLH, MYB, WRKY, etc. They can also regulate plant tolerance to low temperature by binding to the promoters of related genes (Sun et al., 2018; Xie et al., 2018; Diao et al., 2020; Li et al., 2020b; Ritonga et al., 2021). In addition, hydrogen peroxide (H_2_O_2_), superoxide anion (O_2_-), and hydroxyl radicals (OH-) in the reactive oxygen species (ROS) signal can activate the MAPK cascade, transcription factors, and redox reactive proteins, thereby participating in plant responses to cold stress (Davletova et al., 2005; Colcombet and Hirt, 2008; Xu et al., 2019). Although the molecular mechanism of cold stress in other plants has been preliminarily analyzed, the molecular genetics analysis of cotton’s low temperature tolerance lags far behind model plants such as *Arabidopsis* and rice. *GhNHL69* is co-expressed with various transcription factors related to cold stress, leading to the *GhNHL69*-silenced plants having more severe dehydration and damage. *GhNHL69* may be related to the expression of abiotic stress-related genes, thereby altering cotton’s cold tolerance (Guo et al., 2023). Overexpression of *GhKCS13* can alter sphingolipids and glycerides of leaves and the fluidity of cell membrane JA synthesis in chloroplasts, thereby creasing the sensitivity of cotton plants to cold stress (Wang et al., 2020a). After *GhCBF4* and *GhZAT10* were silenced by virus-induced gene silencing (VIGS), the silent plants exhibit significant low temperature sensitivity (Li et al., 2023a).

At present, based on a single temperature stress, researchers have discovered some genes and pathways related to cold stress in cotton (Cheng et al., 2020; Kaur Dhaliwal et al., 2021; Wang et al., 2021). However, the mechanism by which cotton perceives and transmits low temperature signals, thereby activating transcription factors and responding to low temperature, is still unclear. There is limited understanding of the functions of key genes that can respond to different cold stresses. The analysis of cotton under cold stress based on RNA seq research methods can help to explore cold resistance genes, elucidate the regulatory mechanisms of low temperature response, study cold resistance mechanisms, and select cold-resistant varieties (Wang et al., 2009; Wang et al., 2020b). Our research group has found that Zhongmian 36 (ZM36) is a cold-tolerant variety (Ma, 2023). Therefore, this study analyzed the changes in the photosynthetic physiological indicators of ZM36 and explored the regulatory pathways and key genes involved in different responses to cold stress by transcriptome sequencing technology (RNA seq). It can provide a more comprehensive understanding of the molecular mechanisms underlying cotton’s response to cold stress.

## 2 Materials and methods

### 2.1 Plant materials and experimental design

ZM36 is provided by the Cotton Molecular Breeding Laboratory of Shihezi University. Cotton seedlings are cultured in an artificial climate box. These are cotton seeds that have undergone germination in a nutrient bowl. The substrate is peat and vermiculite, with a ratio of 3:1. The plants were cultivated under 24/22°C (day/night) and a photoperiod scheme of 16/8 h of light/darkness. Seedlings at the two-leaf stage were processed at low temperature in an incubator with adjustable temperature settings.

The processing method is as follows: cotton seedlings with consistent growth are grown for 24 h at 25°C (T25, control), 15°C (T15), 10°C (T10), and 4°C (T4), then sampled, frozen, and stored at -80°C. The experiment setup had three replicates, with five identical cotton seedlings as one replicate, and each treatment setup had three biological replicates.

### 2.2 Photosynthetic performance index and fluorescence parameters

Portable photosynthetic instrument LI-6400XT (LI-COR, USA) is used to measure the photosynthetic performance parameters, with the instrument’s built-in red and blue light source selected and the light intensity set to 1,000 μ Mol m^-2^ s^-1^. HandyPEA-100 (UK) was used to measure the fluorescence parameters, and these were measured at the same leaf position of seedlings with similar growth. The conductivity meter method was used to measure the relative conductivity, and the acidic ninhydrin colorimetric method was employed to measure the proline content.

### 2.3 RNA library construction and sequencing

RNA Purification Kit (Tiangen, Beijing) was used to isolate RNA from the leaves of 12 samples (ZM36) according to the manufacturer’s instructions. The RNA isolated from each sample was then used to construct RNA-seq libraries using NEBNext Ultra RNA Library Prep Kit. RNA-seq was conducted on an Illumina Hiseq 4000 platform with 150-bp paired-end reads (Novogene, Tianjin, China).

### 2.4 Quantitative RT-PCR analysis

qRT-PCR was carried out by using SYBR Green (Roche, Rotkreuz, Switzerland) on Light Cycler 480II (Roche) with default parameters. All primers used for the validation experiments were designed with Primer5 software and are shown in **Supplementary Table S1**. The *GhUBQ7* (DQ116441.1) gene served as an internal control to normalize differences between samples. The qRT-PCR conditions were as follows: initial denaturation at 96°C for 5 min, denaturation at 96°C for 15 s for a total of 41 cycles, annealing at 62°C for 16 s, and extension at 70°C for 18 s. The relative expression levels of genes from three biologically independent experiments were calculated using the 2^-ΔΔCT^method (Livak and Schmittgen, 2001).

### 2.5 Identification and functional annotation of DEGs

Clean reads were mapped to the reference genome of *G. hirsutum* (Hu et al., 2019) using TopHat (v2.0.12). The level of gene expression was measured by fragments per kilobase of exon model per million mapped fragments (FPKM) (Trapnell et al., 2010). DESeq2 (v1.18.0) (Wang et al., 2010) was used to identify differentially expressed genes (DEGs) with the criteria of an adjusted log_2_ (fold change) ≥3. The STEM software was used to classify the gene expression patterns at various points into differential clusters. Using the cluster Profiler package (version 3.18.1), DEG enrichment analysis was conducted using Gene Ontology (GO) and Kyoto Encyclopedia of Genes and Genomes (KEGG).

### 2.6 Construction of DEG PPI protein interaction network

We used Blast (blast x) for the sequences of DEGs with the genomes of related species to obtain the predicted PPI for these DEGs (the protein interactions exist in the STRING database: http://string-db.org/. Then, the PPI of these DEGs was visualized using Cytoscape (Shannon et al., 2003).

### 2.7 Virus-induced gene silencing

Tobacco rattle virus (TRV) vectors, pTRV1 and pTRV2, were used in the VIGS experiments, and TRV:: *GhCHLI* was used as a positive control as previously reported (Li et al., 2023b). A 300-bp fragment specific to *GhPLATZ* (*GH_A09G2161*) was amplified by PCR from low-resistance ZM36 with gene-specific primers (**Supplementary Table S2**). VIGS was performed with the same procedures as previously described (Gao et al., 2013). Approximately 12 days after infiltration, the leaves of five TRV::*00* and TRV:: *GhPLATZ* plants were collected to analyze the expression level of *GhPLATZ* by qRT-PCR. A total of 60 TRV::*00* and TRV:: *GhPLATZ* plants at the two-leaf-stage were subjected to low temperature treatment to compare their phenotypic response.

## 3 Results

### 3.1 Photosynthetic physiology of ZM36 under cold stress

**Figure 1:**
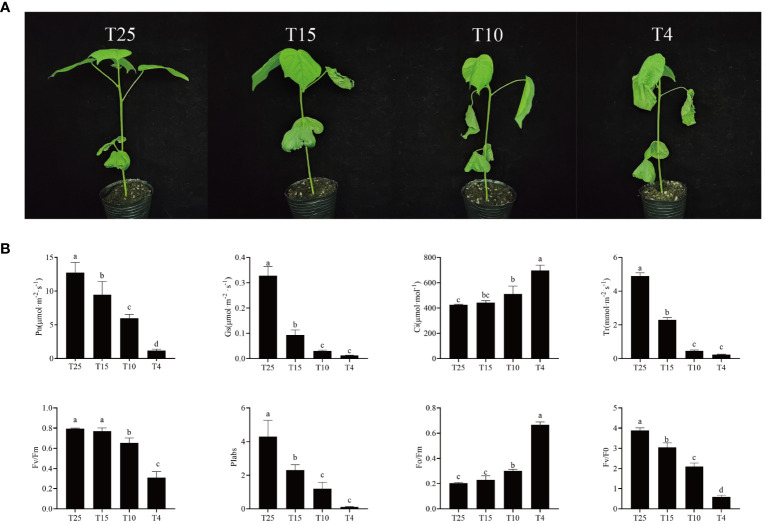
Phenotype and photosynthetic level of ZM36. **(A)** Phenotype of ZM36 in four temperature points (T25, T15, T10, and T4). **(B)** Photosynthetic gas exchange parameters and rapid chlorophyll fluorescence parameters of ZM36 in four temperature points (T25, T15, T10, and T4). Data are the mean ± standard error of three independent biological replicates. Different lowercase letters (a, b, c) indicate a significant difference (*P* < 0.05) between groups determined using Student’s *t*-test.

ZM36 was tested for Pn, Gs, Ci, Tr, Fv/Fm, Piabs, Fv/Fo, and Fo/Fm in four different temperature points (T25, T15, T10, and T4) (**Figure 1A**). Pn, Gs, Tr, Fv/Fm, Fv/Fo, and Piabs significantly decreased, but Ci and Fo/Fm significantly increased under cold stress. The highest value of Pn is 12.75 at T25, the minimum is 1.18 at T4, reduced by 10.8 times; Gs decreased by 26.28 times from T25 (0.3278) to T4 (0.0124); Tr decreased by 20.75 times from T25 (4.9022) to T4 (0.2362); Fv/Fm was 0.7951 and 0.311 at T25 and at T4, respectively, reduced by 2.56 times; and Piabs decreased by 37.81 times from T25 (4.2996) to T4 (0.1137). However, Ci increased by 1.64 times from T25 to T4, and Fo/Fm also increased by 3.25 times from T25 (0.2049) to T4 (0.6659) (**Figure 1B**; **Supplementary Table S2**). This fully demonstrates that low temperature can have a significant impact on cotton growth, photosynthesis, and yield of cotton, thereby affecting its yield and quality.

### 3.2 Identification of DEGs in response to cold stress

**Figure 2:**
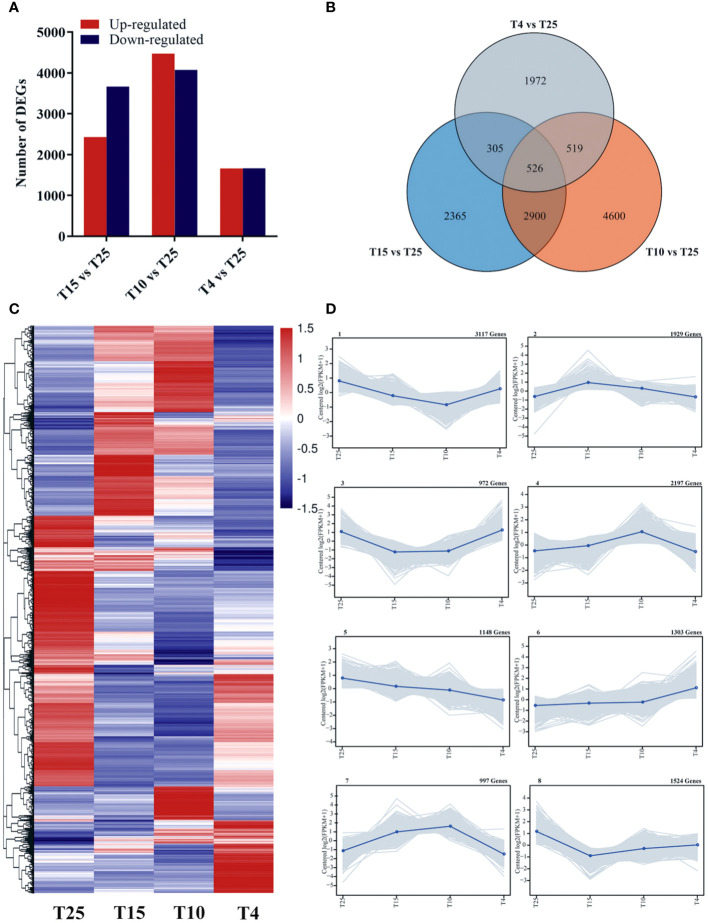
Number of differentially expressed genes (DEGs) upon cold stress at three temperature points (T15 vs. T25, T10 vs. T25, and T4 vs. T25). **(A)** Number of upregulated and downregulated genes at three temperature points (T15 vs. T25, T10 vs. T25, and T4 vs. T25). **(B)** Venn diagram showing the number of DEGs at three temperature points (T15 vs. T25, T10 vs. T25, and T4 vs. T25). **(C)** Heat map of DEGs at four temperature points (T25, T15, T10, and T4). **(D)** Trend analysis of the co-expression patterns of DEGs at four temperature points (T25, T15, T10, and T4).

The number of raw reads of the samples was between 40,476,800 and 49,397,772, and the number of clean reads after filtering was 20,238,400–24,698,886 (**Supplementary Table S3**). The GC percentage of each sample was between 43.46% and 44.34%, while Q30 was between 90.16% and 95.04% (**Supplementary Table S3**). Pearson correlation coefficient was used to perform a correlation test on the samples, and the correlation between the three replicates was greater than 0.9, indicating the reliability of the data (**Supplementary Figure S1A**). Principal component analysis (PCA) was used to test the similarity between samples. The first principal component (PC1) accounted for 43%, while the second principal component (PC2) accounted for 26.3% (**Supplementary Figure S1B**). The volatility of log10^FPKM^ is similar across all samples (**Supplementary Figure S1C**), indicating that the sequencing quality was high and suitable for subsequent analysis. Using fold change ≥3 and FDR <0.05 as the threshold, 6,096 DEGs (2,430 upregulated and 3,666 downregulated), 8,545 DEGs (4,473 upregulated and 4,072 downregulated), and 3,322 DEGs (1,659 upregulated and 1,663 downregulated) were screened in T15 vs. T25, T10 vs. T25, and T4 vs. T25, respectively, indicating that gene expression levels in cotton varied after different low temperature treatments (**Figures 2A, C**). Using Wayne analysis, a total of 13,183 DEGs were found, of which 2,365 DEGs were specifically responsive to 15°C, 4,600 DEGs were specifically responsive to 10°C, and 1,972 DEGs were specifically responsive to 4°C (**Figure 2B**). The DEGs were divided into eight significant clusters by K-means clustering analysis (**Figure 2D**). The genes of clusters 1, 2, and 3 showed a trend of rising first and then remaining unchanged or decreasing slightly in response to low temperature stress. However, the genes of clusters 5, 6 and 7 had an expression trend opposite to that of clusters 1, 2, and 3. The genes of cluster 4 showed a decreasing trend, and cluster 8 had an expression trend opposite to that of cluster 4 (**Figure 2D**). The DEGs of these clusters would be expected to be linked to the low temperature resistance of ZM36.

### 3.3 Validation of differentially expressed genes by qRT-PCR

**Figure 3:**
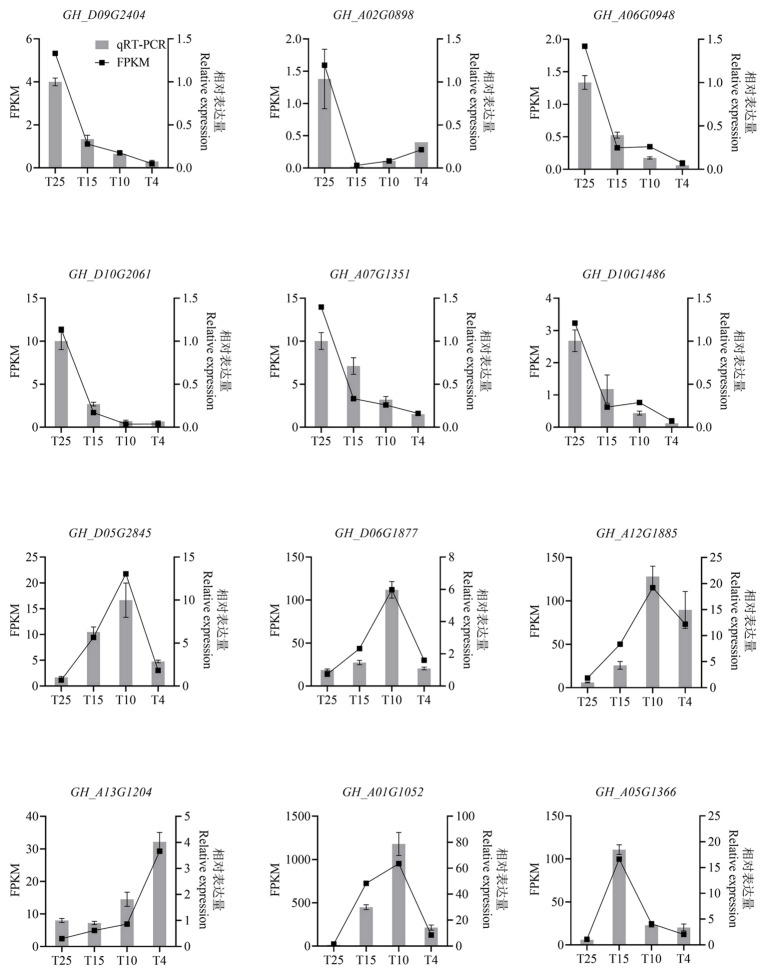
Histogram showing the relative expression level of the 12 selected genes in cotton leaves at the four temperature points after cold stress by qRT-PCR and RNA-seq analysis. The 12 selected genes are six significant downregulation expressed genes (*Gh_D09G2404*, *Gh_A02G0898*, *Gh_A06G0948*, *Gh_D10G2061*, *Gh_A07G1351*, and *Gh_D10G1486*), six significant upregulation expressed genes (*Gh_D05G2845*, *Gh_D06G1877*, *Gh_A12G1885*, *Gh_A13G1204*, *Gh_A01G1052*,and *Gh_A05G1366*). FPKM, fragments per kilobase of exon model per million mapped fragments.

The expression patterns of 12 genes were validated using qRT-PCR, including six significant downregulation expressed genes (*Gh_D09G2404*, *Gh_A02G0898*, *Gh_A06G0948*, *Gh_D10G2061*, *Gh_A07G1351*, and *Gh_D10G1486*), six significant upregulation expressed genes (*Gh_D05G2845*, *Gh_D06G1877*, *Gh_A12G1885*, *Gh_A13G1204*, *Gh_A01G1052*, and *Gh_A05G1366*). All these genes showed the same expression trend between qRT-PCR (**Figure 3**) and RNA-Seq (**Figure 3**) at T25, T15, T10, and T4, confirming the reliability of the RNA-seq result.

### 3.4 Gene ontology analysis of DEGs

**Figure 4:**
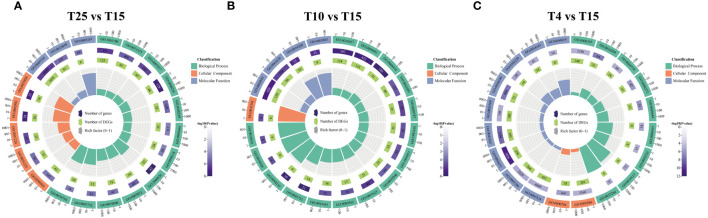
Gene ontology enrichment analysis for the differentially expressed genes identified in T15 vs. T25, T10 vs. T25, and T4 vs. T25. **(A)** Enriched GO terms at T15 vs. T25 in ZM36. **(B)** Enriched GO terms at T10 vs. T25 in ZM36. **(C)** Enriched GO terms at T4 vs. T25 in ZM36. Count means the number of genes included in the GO term.

GO enrichment analysis of DEGs was performed to determine the functions of the distinct transcripts differentially expressed in ZM36 after low temperature stress. In T15 vs. T25, the GO terms such as response to ATP binding (GO:0005524), response to oxygen-containing compound (GO:1901700), cell wall organization (GO:0071555), microtubule-based process (GO:0007017) were commonly enriched (**Figure 4A**). The GO terms such as response to ubiquitin–protein transferase activity (GO:0004842), protein ubiquitination (GO:0016567), response to external stimulus (GO:0009605), ubiquitin protein ligase activity (GO:0061630), and response to water deprivation (GO:0009414) were commonly enriched in T10 vs. T25 (**Figure 4B**). In T4 vs. T25, the DEGs are mainly enriched in ATP binding (GO:0005524), plasma membrane (GO:0005886), transcription factor activity, sequence-specific DNA binding (GO:0003700), protein kinase activity (GO:0004672), and regulation of transcription, DNA-templated (GO:0006355) (**Figure 4C**).

### 3.5 KEGG pathway analysis of DEGs

**Figure 5:**
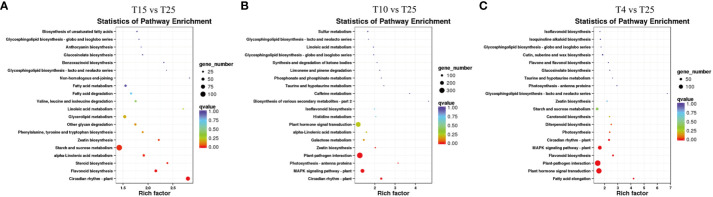
KEGG analysis of differentially expressed genes (DEGs) associated with the response to cold stress in T15 vs. T25, T10 vs. T25, and T4 vs. T25. **(A)** KEGG categories of DEGs in T15 vs. T25. **(B)** KEGG categories of DEGs in T10 vs. T25. **(C)** KEGG categories of DEGs in T4 vs. T25.

The KEGG enrichment analysis of all the DEGs resulted in 2,454 pathways. There were many significant changes in pathways related to circadian rhythm—plants, beta-alanine metabolism, zeatin biosynthesis, fatty acid metabolism, biosynthesis of amino acids, MAPK signaling pathway–plant, glutathione metabolism, and plant–pathogen interaction (**Figures 5A–C**), which are mainly associated with plant growth and development and response to stresses. The KEGG pathways enriched in T4 vs. T25 included MAPK signaling pathway–plant, plant–pathogen interaction, photosynthesis–antenna proteins, photosynthesis, flavonoid biosynthesis, and plant hormone signal transduction (**Figure 5C**). The enriched pathways also suggested that genes related to MAPK cascade signaling might be related to the cotton response to low-temperature stress. The pathways related to MAPK signaling pathway–plant were enriched in two temperature points (T10 and T4) (**Figures 5B, C**). The genes involved included those encoding MAPKKK18 (*GH_A03G0386*), serine/threonine protein kinase OXI1 (*GH_A07G2243*), abscisic acid receptor PYR1 (*GH_A12G2288*), ultraviolet-B receptor UVR8 (*GH_A05G3668*), and PLATZ transcription factor family protein (*GH_A09G2161*). The above-mentioned results indicated that the response of cotton to low temperature stress is governed by a complex gene network that regulates multiple metabolic pathways.

### 3.6 Transcription factor prediction

In T15 vs. T25, T10 vs. T25, and T4 vs. T25 groups, 471 transcription factors (49 transcription factor families), 767 transcription factors (59 transcription factor families), and 341 transcription factors (41 transcription factor families) were found to be involved in the response to different degrees of cold stress, respectively. To predict the transcription factors of all DEGs, 1,119 transcription factors were found in 59 transcription factor families (**Supplementary Figure S2A**). Among them, different members of major gene families such as AP2/ERF-ERF (133 genes), MYB (108 genes), bHLH (78 genes), and NAC (73 genes) were upregulated or downregulated under different cold stresses (**Supplementary Figures S2B–E**).

### 3.7 Phosphatidylinositol signaling system analysis of DEGs

The phosphatidylinositol signaling system regulates many physiological processes, such as growth, cytoskeleton rearrangement, and membrane transport. The DEGs of the phosphatidylinositol signaling system (ko04070) were identified to be 32 genes (21 upregulated, 11 downregulated), 40 genes (29 upregulated, 11 downregulated), and 22 genes (18 upregulated, four downregulated) in T15, T10, and T4, respectively (**Supplementary Figure S3**). Among them, all others were upregulated except for *GH_D10G0541* in two 1-phosphatidylinositol-3-phosphate 5-kinase (FAB1) after cold stress (**Supplementary Figure S3A**). *GH_A10G0515* was upregulated 4.60, 3.88, and 5.66 times after T15, T10, and T4, respectively (**Supplementary Figure S3A**). Six phosphatidylinositol-specific phospholipases (PLCD) were downregulated except for *GH_A06G1884* (**Supplementary Figure S3A**). Three diacylglycerol kinases (DGK) were upregulated under different cold stresses, except for *GH_A12G2233*, and *GH_D12G2002* was upregulated 4.40, 15.03, and 2.40 times after T15, T10, and T4, respectively (**Supplementary Figure S3B**). Most of the 25 calmodulin (CALM) genes were upregulated, especially *GH_A04G1762* and *GH_D12G1967*. They were upregulated by 104.81, 17.55, 4.12 times and 74.50, 76.91, and 2.80 times after T15, T10, and T4, respectively (**Supplementary Figure S3B**).

### 3.8 Photosynthetic-related pathway analysis of DEGs

**Figure 6:**
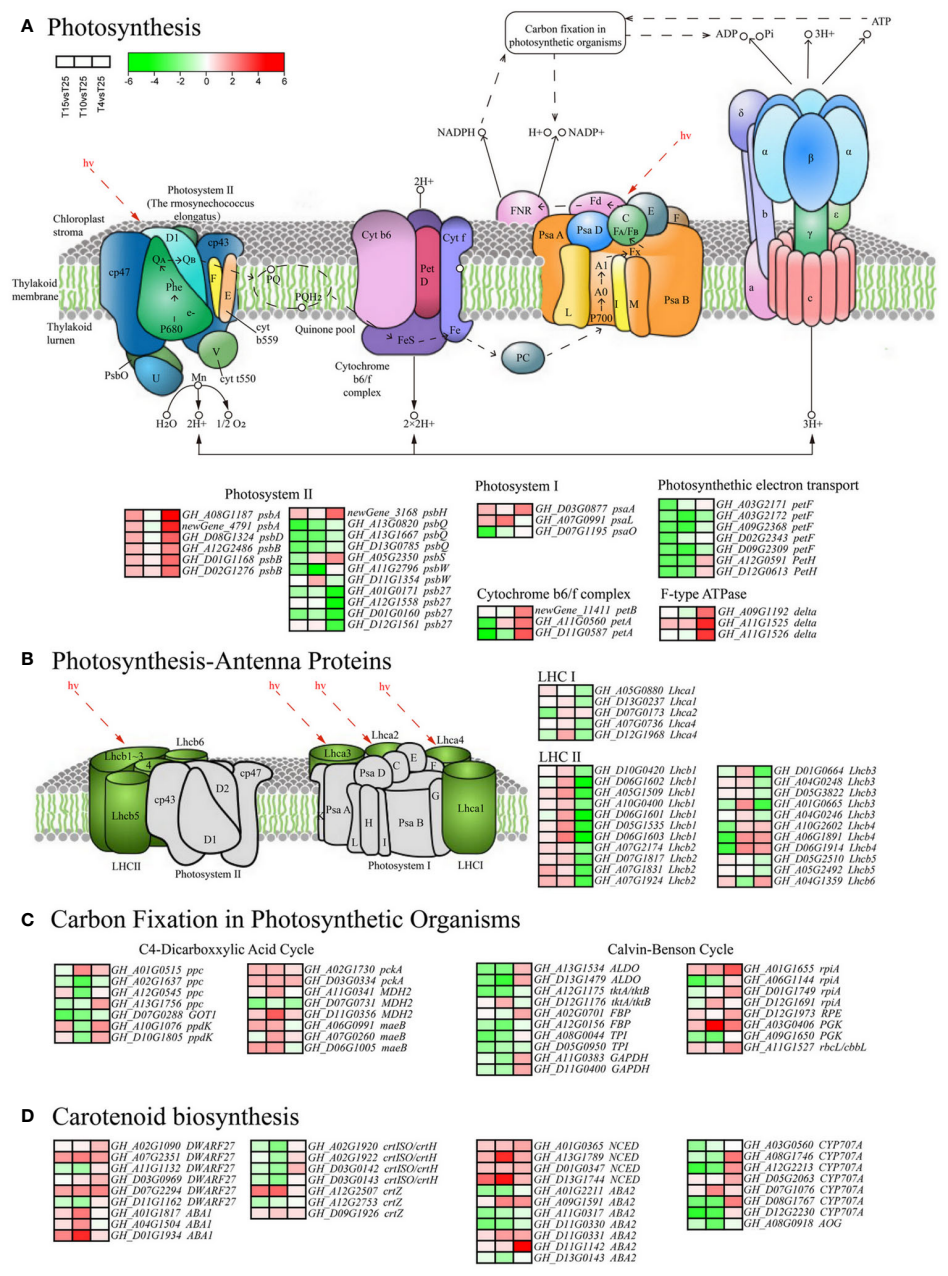
Photosynthetic-related pathway analysis of differentially expressed genes (DEGs). **(A)** Enrichment analysis of 34 DEGs related to photosynthesis (ko00195). **(B)** Enrichment analysis of 27 DEGs (five LHC I and 22 LHC II) related to the photosynthesis antenna proteins (ko00196). **(C)** Enrichment analysis of 34 DEGs related to carbon fixation in photosynthetic organisms (ko00710). **(D)** Enrichment analysis of 35 DEGs related to the carotenoid biosynthesis (ko00906).

Photosynthesis is sensitive to cold stress. KEGG indicate that many DEGs are involved in photosynthesis metabolic pathways. In this study, 34 differentially expressed genes were enriched under different cold stresses in photosynthesis (ko00195) (**Figure 6A**). One psaA, one psaL, and one psaO gene were found in the photosystem I complex. Among them, psaA (*GH_D03G0877*) was upregulated 1.93, 1.20, and 2.99 times after T15, T10, and T4, respectively. Two psbA, one psbD, three psbB, and one psbH gene in the photosystem II complex were upregulated after T15, T10, and T4, while four psb27 genes were downregulated. One petB gene and one petA gene were found in the cytochrome b6-f complex, which were upregulated under T4 for 3.13, 3.52, and 4.61 times, respectively. Seven DEGs related to photosynthetic electron transfer were downregulated after T15, T10, and T4, indicating that cold stress has a significant inhibitory effect on electron transfer in photosynthesis. The 27 DEGs (five LHC I and 22 LHC II) were found in the photosynthesis antenna proteins (ko00196) (**Figure 6B**). Except for three Lhcb4 genes that were downregulated after T15 and T10 and upregulated after T4, most genes were upregulated after T15 and T10 and downregulated after T4. The 24 genes were upregulated after T15 and T10 and downregulated after T4, and three Lhcb4 genes were exactly the opposite. A total of 34 DEGs were found in carbon fixation in photosynthetic organisms (ko00710) pathway (**Figure 6C**). The C4 pathway is enriched to 15, and the Calvin cycle is enriched to 19. Among them, one ribose 5-phosphate isomerase A (*GH_A01G1655*) in the Calvin cycle was upregulated by 2.00, 2.75, and 6.72 times after T15, T10, and T4 stress, respectively, and one phosphoglycerate kinase (*GH_A03G0406*) was upregulated 2.03, 20.07, and 4.49 times after T15, T10, and T4 stress, respectively. At the same time, we also discovered 35 DEGs (**Figure 6D**). Six genes were in carotenoid biosynthesis (ko00906) β. Most of the beta carotene isomerase (DWARF27) was upregulated, especially *GH_A07G2351* and *GH_D07G2294*, which were upregulated 4.99, 8.27, and 4.70 times and 4.06, 6.08, and 7.14 times after T15, T10, and T4 stress, respectively. Beta carotene 3-hydroxylase (crtZ) was upregulated in expression. Three zeaxanthin epoxidase (ZEP) genes were upregulated, with *GH_D01G1934* upregulated 7.35, 40.20, and 1.51 times after T15, T10, and T4 stress, respectively. Four 9-cis-epoxycarotene dioxygenase (NCED) genes were upregulated, with *GH_D13G1744* upregulated 12.84, 58.35, and 2.99 times after T15, T10, and T4 stress, respectively.

### 3.9 Analysis of co-expressed DEG interactions

**Figure 7:**
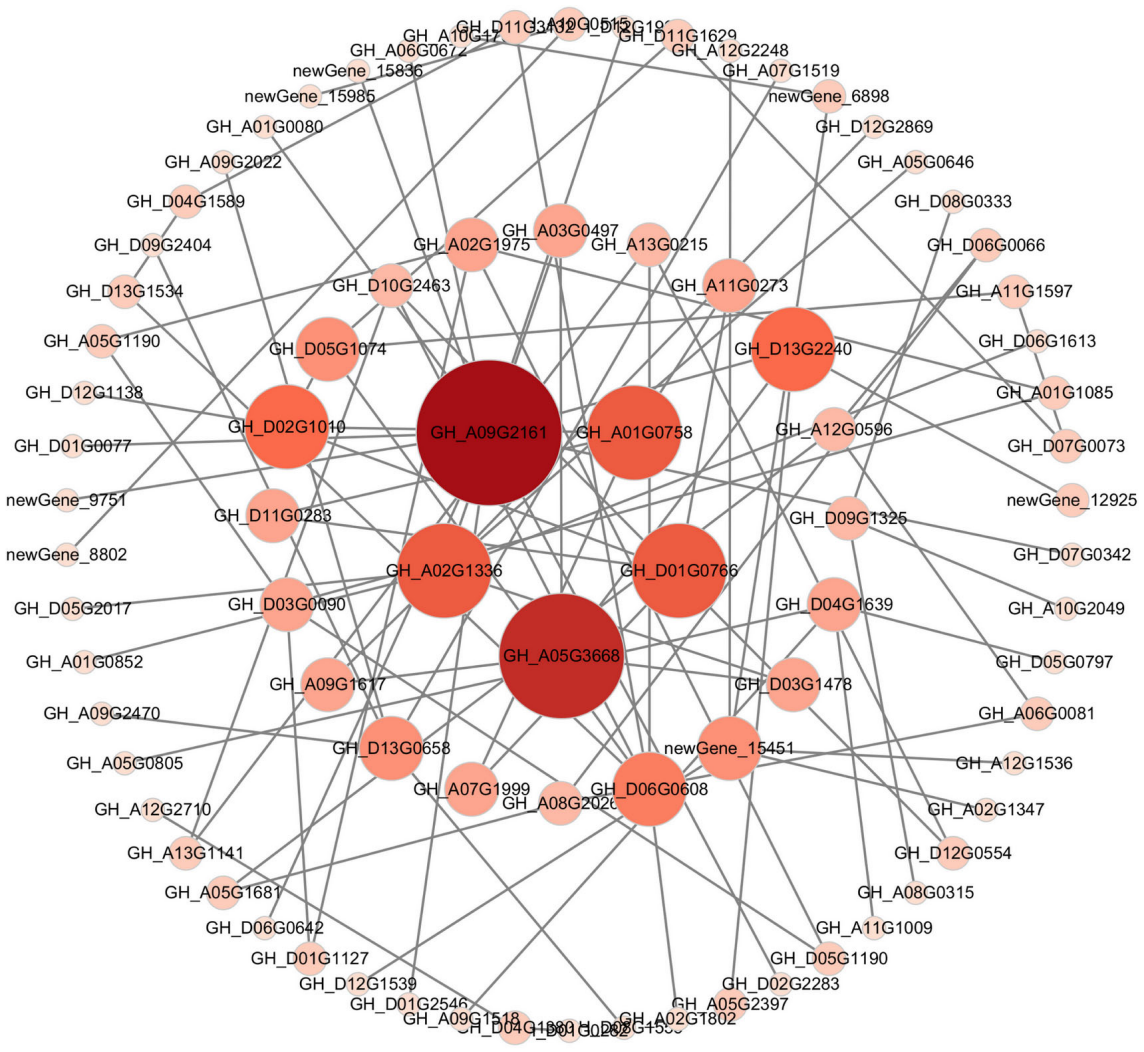
Co-expression network analysis results of the hub genes. Each circle represents a hub gene. Circle size and col vs. or represent the degree.

The 526 DEGs were detected under three different cold stress conditions, indicating that the DEGs may have been involved in adapting to stress at different low temperature points, and participating in the same pathway. Therefore, we defined 526 co-expressed DEGs as key cold-resistant genes. Further utilizing the STRING database to predict the interrelationships between 526 proteins, Cytoscape software was used for visualization processing, and five hub genes were identified based on the criteria of degree value ≥16 (**Figure 7**). Four genes were significantly upregulated under cold stress (*GH_D01G0766*, *GH_A05G3668*, *GH_A01G0766*, and *GH_A09G2161*), while one gene was significantly downregulated under cold stress (*GH_A02G1336*).

### 3.10 Silencing of *GhPLATZ* reduces the resistance of cotton to cold stress

**Figure 8:**
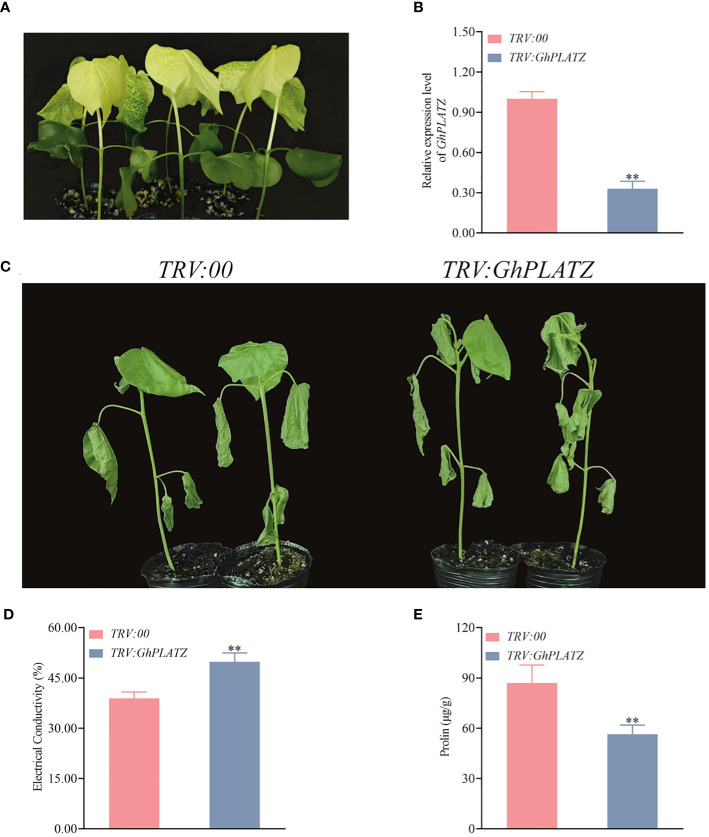
Knockdown of *GhPLATZ* in cold stress-resistant ZM36 reduced low temperature point resistance. **(A)** Observation of the expected yellowing leaf phenotype in TRV:: *GhCHLI* plants. **(B)** *GhPLATZ* expression in the TRV:*00* and TRV:: *GhPP2C52* plants. **(C)** Cold phenotypes of the TRV:: 00 and TRV:: *GhPLATZ* plants at 10 day. **(D)** Comparison of electrical conductivity from the TRV:: *00* and TRV:: *GhLATZ* plants at 10 day. **(E)** Comparison of proline between TRV:*00* and TRV:: *GhPLATZ* plants at 10 day. The data are three independent biological replicates, Significance analysis using T test (** P < 0.01).

To get insight on the potential function of *GhPLATZ*, we knocked down the expression level of *GhPLATZ* in ZM36 using VIGS with cotton seedlings treated by TRV:: *GhCHLI* as positive control of the VIGS experiment (**Figure 8A**). Compared to TRV2::00 plants, TRV:: *GhPLATZ* plants had a significantly low expression level of *GhPLATZ* (**Figure 8B**; **Supplementary Table S4**), suggesting the successful inhibition of *GhPLATZ* by VIGS. After about 10 days of cold stress, the TRV:: *GhCHLI* plants showed a yellowing phenotype (**Figure 8A**), indicating that the VIGS system was functioning properly. To confirm the effectiveness of the silencing system, cotton seedlings were subjected to 15°C (low temperature treatment) to verify the resistance of cotton to low temperature stress after inhibiting *GhPLATZ* expression. After 48 h of treatment at 15°C, it was found that TRV:: *GhPLATZ* plants were more sensitive to low temperature compared to TRV::00 plants, with more significant leaf wilting and dehydration (**Figure 8C**; **Supplementary Table S4**), indicating that inhibiting the expression of *GhPLATZ* would reduce cotton’s resistance to low temperature. The electrical conductivity of TRV : *GhPLATZ* plants was 49.82% ± 0.03, significantly higher than that of TRV:*00* plants (**Figure 8D**; **Supplementary Table S4**). Consequently, the proline of TRV : *GhPLATZ* plants was higher than that of TRV:*00* plants at 87 and 56 ug^-1^ g, respectively (**Figure 8E**; **Supplementary Table S4**).

## 4 Discussion

### 4.1 Changes in photosynthetic characteristics under cold stress

Cold stress can induce photoinhibition, reducing the absorption and capture capacity of the light system, electron transfer efficiency, and fixed quantum efficiency of carbon dioxide. In this study, the photosynthetic system was significantly damaged, inhibiting the normal progress of photosynthesis and leading to the generation of photoinhibition effects. Especially under 4°C, the photoinhibition effect was more severe. The downregulation of LHCA and LHCB genes under cold stress reduces the ability to absorb and capture light energy (Wilson et al., 2006). The downregulation of key enzyme activity and gene transcriptional expression levels in the Calvin cycle may be the reason for the decrease in plant carbon assimilation efficiency and RuBP regeneration rate caused by low temperature (Hussain et al., 2021). In this study, 27 DEGs were enriched in the photosynthesis antenna protein pathway under cold stress, while LHCA and LHCB genes were upregulated, which may be an adaptive protective response. The 34 DEGs are involved in the carbon fixation pathway in photosynthetic organisms, of which 19 are related to the Calvin cycle. The DEGs related to photosynthetic electron transfer are downregulated, indicating severe damage to the photosynthetic system, and reduce the efficiency of carbon assimilation under cold stresses. However, some DEGs were upregulated, although the Pn and Fv/Fm values decreased in T4. It is speculated that ZM36 can resist low temperature stress and alleviate oxidative damage by regulating the expression of photosynthetic-related genes. In addition, proline is a common osmoregulatory substance in plants, which can maintain the osmotic balance between the protoplast and the environment, thereby alleviating the damage caused by low temperature. Plants accumulate higher levels of proline when subjected to stress (Charest and Ton Phan, 1990; Shen et al., 2020). This study found that the proline content in silenced plants decreased by 30.1% compared to the control plants after low temperature stress, indicating a significant decrease in the cold tolerance of silenced plants. This suggests that enhancing the expression of photosynthesis-related genes is not enough to maintain a certain level of photosynthetic ability under cold stress. The specific mechanism values need further in-depth research.

### 4.2 Transcription factors AP2/ERF, MYB, and NAC regulate cotton cold tolerance

When plants are subjected to cold stress, transcription factors (TFs) bind to specific *cis* regulatory elements in the promoter to regulate target genes related to cold resistance, thereby enhancing cold tolerance (Knight and Knight, 2012; Zhao et al., 2015). In recent years, TF families such as AP2/ERF, MYB, bHLH, and NAC have received widespread attention as key regulatory factors in plant stress response (Mehrotra et al., 2020; He et al., 2023). AP2/ERF plays a crucial regulatory role in response to low temperature stress. In addition, AtMYB15 plays a negative regulatory role in regulating cold resistance in *Arabidopsis* (Wang et al., 2019). The NAC family is the largest specific transcription factor family in plants, playing a crucial regulatory role in plant growth, development, and response to abiotic stress (Tran et al., 2010). In *Arabidopsis*, overexpression of HuNAC20 and HuNAC25 enhances tolerance to cold stress by altering the expression of cold response genes in transgenic plants (Hu et al., 2022). In this study, the number of members belonging to the AP2/ERF transcription factor family was the highest. At the same time, MYB transcription factors also play a crucial role in low temperature response. Most members of the MYB family show a downward trend under low temperature stress, and most genes in the NAC family show varying degrees of upregulation under different low temperature stress conditions, such as *GH_D02G1383* and *GH_A03G1198*, which were upregulated hundreds of times under low temperature stress, highlighting the important role of the NAC family in cotton’s resistance to low temperature stress.

### 4.3 Key genes for cold resistance and functional validation of *GhPLATZ*

Cold resistance is a complex physiological and biochemical process involving gene regulation. Plants improve cold resistance by coordinating the expression of multiple genes (Umer et al., 2020) UVR8 is a photoreceptor that specifically absorbs UV-B light (Kliebenstein et al., 2002). The bZIP transcription factor HY5 is activated by UVR8 binding to COP1, thereby inducing the expression of multiple metabolic pathway genes (Favory et al., 2009). The tomato UVR8 gene participates in UV-B-induced cold tolerance by upregulating CuZnSOD, FeSOD, and CAT1 genes (Jiang et al., 2022). UVR8 regulates plant response to UV-B light by interacting with various transcription factors such as COP1 and HY5, including light morphogenesis, secondary metabolism, and adaptability to environmental stress. These transcription factors may affect the expression of genes related to ascorbic acid synthesis and metabolism, thereby affecting the level of ascorbic acid in plants (Lin et al., 2020; Podolec et al., 2021). The zinc finger transcription factor PLATZ is widely present in plants and plays an important role in regulating plant growth and development and responding to abiotic stress. The transgenic *Arabidopsis thaliana* with *GhPLATZ1* may promote seed germination and seedling formation under salt stress by increasing the GA and ethylene content and reducing the ABA content (Han et al., 2022). It was found that *PhePLATZ23* and *PLATZ27* are highly responsive to cold stress and play an extremely important role in regulating bamboo’s response to external environmental stimuli (Zhang et al., 2022). UVR8 mainly involves the perception and transduction of light signals, while the PLATZ transcription factor is more related to plant transcriptional regulation and stress response. Although both play important roles in plant life activities, their mechanisms of action and biological functions are different, and there is currently no clear evidence to suggest a direct interaction or functional connection between them. Future research may reveal whether there are some unknown connections between them or whether they have cross-functional pathways in plant life activities. In this study, the five key candidate genes in response to cold stress were identified, including *GhUVR8* (*GH_A05G3668*) and *GhPLATZ* (*GH_A09G2161*). Meanwhile, it was also found that *GhPLATZ* was subjected to low temperature treatment after transient silencing, and the silenced plants were more sensitive to low temperature and suffered more severe damage than the control plants. It is speculated that *GhPLATZ* is positively regulating cotton’s tolerance to low temperature.

## 5 Conclusions

The cold resistance of ZM36 is a complex process that involves the synergistic effects of genes, proteins, and metabolic pathways. These mechanisms interact with each other and together form the strong cold resistance of ZM36, enabling it to maintain relatively normal growth and development in low temperature conditions. In addition, network regulatory analysis identified five hub genes, including *GhPLATZ* (*GH_A09G2161*) highly related to the response of cotton plants to cold stress. *GhPLATZ* was demonstrated to be a positive regulator of low temperature response by VIGS. This discovery provides us with new ideas and methods for further understanding the cold resistance mechanism of plants.

## Data availability statement

The original contributions presented in the study are publicly available. This data can be found at the National Center for Biotechnology Information (NCBI) using accession number PRJNA498759.

## Author contributions

YL: Conceptualization, Formal analysis, Methodology, Visualization, Writing – original draft, Writing – review & editing, Data curation, Investigation, Funding acquisition, Resources. JZ: Data curation, Formal analysis, Investigation, Methodology, Visualization, Writing – original draft, Writing – review & editing. JX: Formal analysis, Writing – review & editing. XZ: Conceptualization, Writing – original draft. ZX: Conceptualization, Data curation, Formal analysis, Investigation, Methodology, Writing – original draft, Resources, Writing – review & editing. ZL: Conceptualization, Data curation, Formal analysis, Funding acquisition, Investigation, Methodology, Writing – original draft, Writing – review & editing, Project administration, Resources.

## Supplementary material

Supplementary Figure 1 Quality analysis of transcriptome sequencing. **(A)** Correlation analysis between 12 samples. **(B)** Principal component analysis between 12 samples. **(C)** Analysis of expression patterns between 12 samples.

Supplementary Figure 2
**(A)** Predictive classification of transcription factors for DEGs. **(B)** Analysis of AP2/ERF-ERF transcription factor expression patterns. **(C)** Analysis of MYB transcription factor expression patterns. **(D)** Analysis of bHLH transcription factor expression patterns. **(E)** Analysis of NAC transcription factor expression patterns.

Supplementary Figure 3 Phosphatidylinositol signaling system analysis of 60 DEGs (FAB1, two; PIP5K, four; PTEN, two; MIMR1_2, 10; PLCD, six; CALM, 25, etc.).

Supplementary Table 1 Primers used for qRT-PCR and VIGS.

Supplementary Table 2 Analysis of eight photosynthetic indicators (Pn, Gs, Ci, Tr, Fv/Fm, Piabs, Fo/Fm, and Fv/Fo) in T25, T15, T10, and T4.

Supplementary Table 3 Transcriptome sequencing data quality and genome mapping.

Supplementary Table 4
*GhPLATZ* expression in the TRV:*00* and TRV:: *GhPLATZ* plants. Analysis of electrical conductivity of TRV:: 00 and TRV:: *GhPLATZ* plants. Analysis of proline of TRV::00 and TRV:: *GhPLATZ* plants.
