# Supplementary figures and images for: Effect of cold stress on photosynthetic physiological characteristics and molecular mechanism analysis in cold-resistant cotton (ZM36) seedlings
Source: Front Plant Sci. 2024 May 13;15:1396666. doi: 10.3389/fpls.2024.1396666 (PMC11128660; doi:10.3389/fpls.2024.1396666)

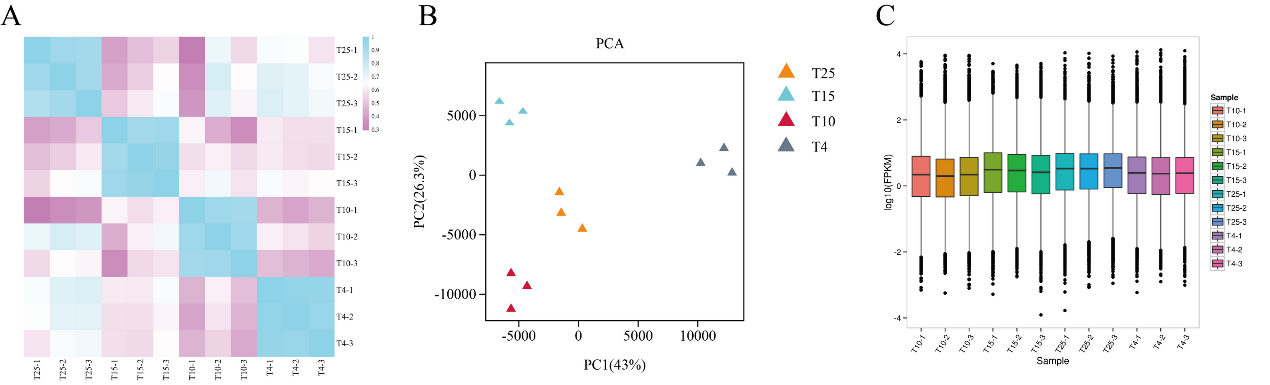
Fig S1:

Fig S2:


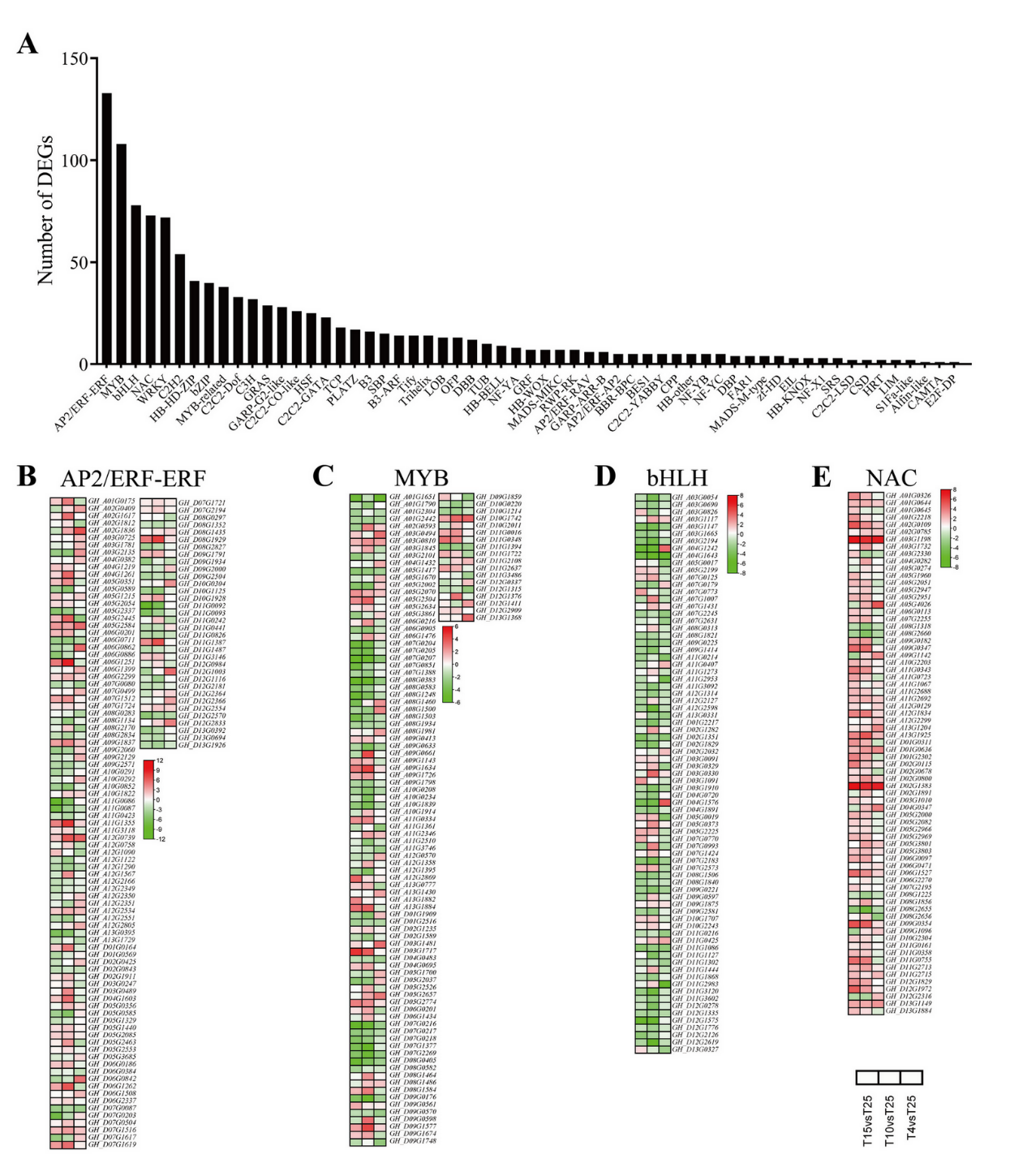


Fig S3:


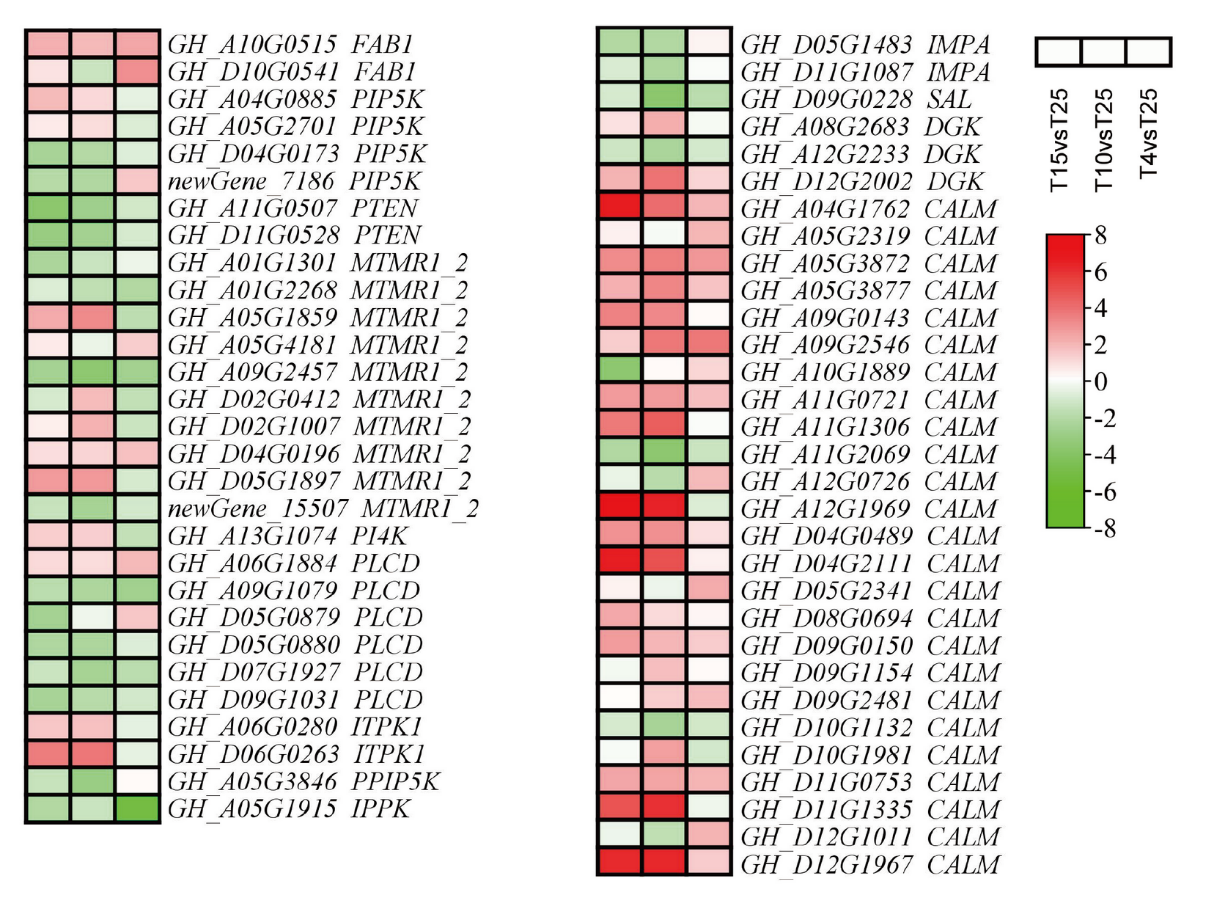

Supplement: Supplementary Figure 1 — Quality analysis of transcriptome sequencing. (A) Correlation analysis between 12 samples. (B) Principal component analysis between 12 samples. (C) Analysis of expression patterns between 12 samples. [file DataSheet_1.docx]
